# Mast Cells Positive for c-Kit Receptor and Tryptase Correlate with Angiogenesis in Cancerous and Adjacent Normal Pancreatic Tissue

**DOI:** 10.3390/cells10020444

**Published:** 2021-02-19

**Authors:** Michele Ammendola, Giuseppe Currò, Carmelo Laface, Valeria Zuccalà, Riccardo Memeo, Francesco Luposella, Mariarita Laforgia, Nicola Zizzo, Alfredo Zito, Donato Loisi, Rosa Patruno, Lucia Milella, Ippazio Ugenti, Mariangela Porcelli, Giuseppe Navarra, Cosmo Damiano Gadaleta, Girolamo Ranieri

**Affiliations:** 1 Department of Health Science, Digestive Surgery Unit, Medical School, University “Magna Graecia”, Viale Europa, Germaneto, 88100 Catanzaro, Italy; michele.ammendola@libero.it (M.A.); currog@unicz.it (G.C.); milellalucia@yahoo.it (L.M.); ippazio.ugenti@uniba.it (I.U.); 2 Interventional Oncology Unit with Integrated Section of Translational Medical Oncology, National Cancer Research Centre, Istituto Tumori “Giovanni Paolo II”, Viale Orazio Flacco 65, 70124 Bari, Italy; carmelo.laface@gmail.com (C.L.); marypor@libero.it (M.P.); c.gadaleta@oncologico.bari.it (C.D.G.); 3 Department of Biomedical Sciences and Clinical Oncology, Section of Oncology, University of Bari ′Aldo Moro′, 70124 Bari, Italy; 4 Pathology Unit, “Pugliese-Ciaccio” Hospital, Viale Pio X°, 88100 Catanzaro, Italy; valezy@libero.it; 5 Department of Emergency and Organ Transplantation, University Aldo Moro of Bari, 70124 Bari, Italy; info@drmemeoriccardo.com; 6 Direction Départementale de la Cohésion Sociale et de la Protection des Populations des VOSGES (DDCSPP88), 88080 Vittel, France; francescoluposella@hotmail.it; 7 Pharmacy Unit, IRCCS Istituto Tumori “Giovanni Paolo II”, Viale Orazio Flacco 65, 70124 Bari, Italy; m.laforgia@oncologico.bari.it; 8 Chair of Pathology, Veterinary Medical School, University “Aldo Moro” of Bari, Via Casamassima, 70010 Bari, Italy; nicola.zizzo@uniba.it (N.Z.); rosavet@libero.it (R.P.); 9 Pathology Unit, National Cancer Research Centre, Istituto Tumori “Giovanni Paolo II”, Viale Orazio Flacco 65, 70124 Bari, Italy; a.zito@oncologico.bari.it (A.Z.); donato.loisi@gmail.com (D.L.); 10 Department of Human Pathology of Adult and Evolutive Age, Surgical Oncology Division, University Hospital of Messina, 98100 Messina, Italy; gnavarra@unime.it

**Keywords:** mast cells, c-Kit receptor, tryptase, angiogenesis, microvascular density, endothelial area, pancreatic cancer tissue, adjacent normal tissue

## Abstract

Background: Mast cells (MCs) contain proangiogenic factors, in particular tryptase, associated with increased angiogenesis in several tumours. With special reference to pancreatic cancer, few data have been published on the role of MCs in angiogenesis in both pancreatic ductal adenocarcinoma tissue (PDAT) and adjacent normal tissue (ANT). In this study, density of mast cells positive for c-Kit receptor (MCDP-c-KitR), density of mast cells positive for tryptase (MCDPT), area of mast cells positive for tryptase (MCAPT), and angiogenesis in terms of microvascular density (MVD) and endothelial area (EA) were evaluated in a total of 45 PDAT patients with stage T_2–3_N_0–1_M_0_. Results: For each analysed tissue parameter, the mean ± standard deviation was evaluated in both PDAT and ANT and differences were evaluated by Student’s *t*-test (*p* ranged from 0.001 to 0.005). Each analysed tissue parameter was then correlated to each other one by Pearson *t*-test analysis (*p* ranged from 0.01 to 0.03). No other correlation among MCDP-c-KitR, MCDPT, MCAPT, MVD, EA and the main clinical–pathological characteristics was found. Conclusions: Our results suggest that tissue parameters increased from ANT to PDAT and that mast cells are strongly associated with angiogenesis in PDAT. On this basis, the inhibition of MCs through tyrosine kinase inhibitors, such as masitinib, or inhibition of tryptase by gabexate mesylate may become potential novel antiangiogenetic approaches in pancreatic cancer therapy.

## 1. Background

Pancreatic cancer (PC) is an infrequent tumour, but at the same time, it corresponds to the fourth most common cause of cancer mortality, with a 5-year survival rate of only 8% and a 10-year survival of just 3%. In its early stages, surgery and adjuvant chemotherapy represent the gold-standard treatment, but nevertheless the relapse rate is 60–70% after 2 years. In the metastatic setting, the FOLFIRINOX regimen is the common first-line treatment for fit patients (Eastern Cooperative Oncology Group Performance Status 0–1 and aged ≤ 75 years), improving median progression-free survival (mPFS) compared to gemcitabine alone (6.4 months vs 3.3 months; *p* < 0.0001) and median overall survival (mOS) up to 11.6 months (versus 6 months for the gemcitabine-alone group; *p* = 0.001) [1]. A multicentre, double-blind phase III study evaluated 154 patients with metastatic PC and BRCA 1–2 germline mutation without disease progression during at least 4 months of first-line platinum-based chemotherapy. This study showed that the median PFS was longer in the olaparib group than in the control group (median 7.4 months versus 3.8 months, respectively; *p* = 0.004) [2].

Computed tomography (CT) diagnoses PC with sensitivity and specificity from 70% up to 100%, and therefore it is always indicated in suspected PC [3]. In patients with clinical and radiological suspicion of PC, the preoperative diagnosis (histological or cytological) should be considered in the absence of clear signs of malignancy and in patients not eligible for surgery. From a pathological point of view, pancreatic ductal adenocarcinoma is the most frequent histotype (85%); it derives from the pancreatic ductal tree and has a glandular differentiation. According to the World Health Organization (WHO), pancreatic ductal adenocarcinoma has been classified into eight variants: colloid carcinoma, signet-ring cell carcinoma, adenosquamous carcinoma, undifferentiated carcinoma with osteoclast-like giant cells, undifferentiated carcinoma, and medullary, rhaboid and hepatoid carcinomas.

Mast cells (MCs) are bone marrow-derived cells, which are found in many human organs and tissues and contain a lot of pre-existing and de-novo-formed secretory granules with peculiar pleiotropic functions [4]. MC activity is mainly regulated by its membrane tyrosine kinase receptor, the c-Kit receptor (c-Kit-R), identified and classified in the cluster of differentiation 117 (CD-117) and binding the stem cell factor (SCF) as its natural ligand [5]. MCs can be activated by several stimuli, including the binding between immunoglobulin E and its antigenic epitope and the interaction between c-Kit-R and SCF [6,7]. After their activation, MCs release their secretory granules in the microenvironment, involved both in allergic reaction and anaphylaxis and in induced immunity [8,9]. In the last decades, several studies have demonstrated that mast cells contain several proangiogenic factors, such as vascular endothelial growth factor (VEGF), fibroblast growth factor-2 (FGF-2) and platelet-derived endothelial cell growth factor/thymidine phosphorylase (PDEC-GF/TP), but their ability to synthesize and secrete a non-classical potent proangiogenic factor called tryptase has only recently become a focus of researchers’ attention [10,11,12]. Tryptase is the most abundant factor stored in mast cell secretory granules. It is a serine protease that has been demonstrated to enhance [13,14,15] endothelial cell (EC) and microvascular proliferation in animal models in several in vitro and in vivo studies [16,17]. Through its binding to protease-activated receptor-2 (PAR-2) on endothelial cells, tryptase can stimulate microvascular formation [18,19,20,21].

Recent preliminary data indicate that mast cells are so involved in pancreatic cancer development that they can even be a therapeutic target, but in this context, very little data have been published so far, as is also the case for the relationship between mast cells density and angiogenesis, including their differences in both primary pancreatic ductal adenocarcinoma tissue (PDAT) and adjacent normal tissue (ANT) [22,23,24,25,26,27,28,29]. MC infiltration is higher in PDAT than in ANT [30,31], and increased MC infiltration directly correlates with higher tumour grade and worse prognosis [32,33]. MCs contribute to the PDAT progression through different mechanisms. For example, MCs express proangiogenic factors such as VEGF, FGF-2 and PDGF, and MC-derived MMPs promote the release of extracellular matrix-bound angiogenic factors. On the other hand, pancreatic cancer cells (PCCs) and stellate pancreatic cells (PSCs) recruit and activate MCs [34]. MCs release IL-13 and tryptase, inducing activation and proliferation of PCCs and PSCs [35]. Moreover, activated PSCs produce several proangiogenic factors and stimulate PCCs [36].

In this exploratory study, through immunohistochemistry and image analysis system, we have examined the density of mast cells positive for c-Kit receptor (MCDP-c-KitR), density of mast cells positive for tryptase (MCDPT), area of mast cells positive for tryptase (MCAPT), microvascular density (MVD) and endothelial area (EA) in both PDAT and ANT, in a series of pancreatic cancer patients who had undergone radical surgery. The correlation between the studied parameters and the main clinical–pathological features has been also investigated.

## 2. Results

**Figure 1:**
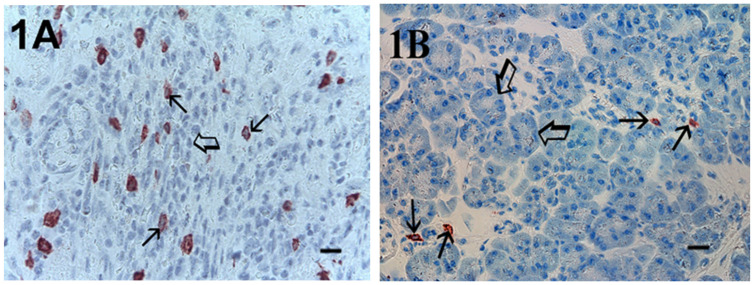
Magnification ×400, 0.19 mm^2^ area, immunostaining with the anti-CD117 antibody. (**A**) High MCDP-c-KitR in primary PDAT section. Small arrows indicate single red-stained MCs. The big arrow indicates tumour epithelium. (**B**) Low MCDP-c-KitR in ANT section. Small arrows indicate single red-stained MCs. Big arrows indicate normal pancreatic epithelium. Scale bar corresponds to 125 µm. MCDP-c-KitR, density of mast cells positive for c-Kit receptor; PDAT, pancreatic ductal adenocarcinoma tissue; MCs, mast cells; ANT, adjacent normal tissue.

**Figure 2:**
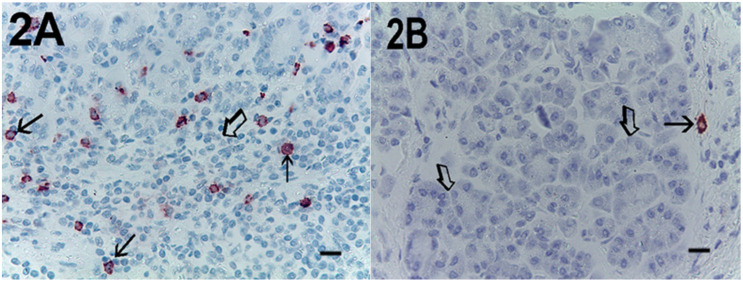
Magnification ×400, 0.19 mm^2^ area, immunostaining with the anti-tryptase antibody. (**A**) High MCDPT in primary PDAT section. Small arrows indicate single red-immunostained MCs. The big arrow indicates tumour epithelium. (**B**) Low MCDPT in ANT section. The small arrow indicates just one red-stained MC. Big arrows indicate normal pancreatic epithelium. Scale bar corresponds to 125 µm. MCDPT, density of mast cells positive for tryptase.

**Figure 3:**
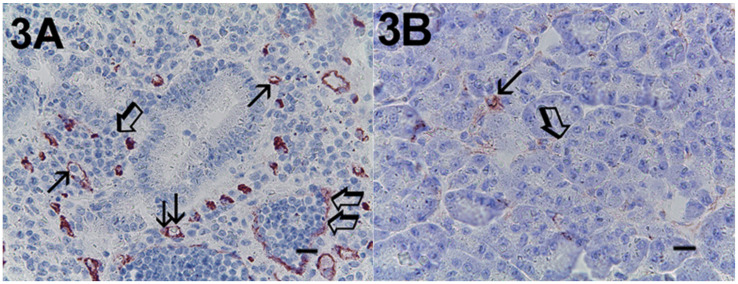
Magnification ×400, 0.19 mm^2^ area, immunostaining with the anti-CD31 antibody. (**A**) High MVD in primary PDAT section. Single small arrows indicate single red-stained microvessels. Double small arrows indicate a single red-stained microvessel with a red blood cell in its lumen as a positive internal control. Single big arrows indicate tumour epithelium. Double big arrows indicate a vessel with a lot of tumour cells in its lumen. (**B**) Low MVD in ANT section. Small arrows indicate a single red-stained microvessel. The big arrow indicates normal pancreatic epithelium. Scale bar corresponds to 125 µm. MVD, microvascular density.

**Figure 4:**
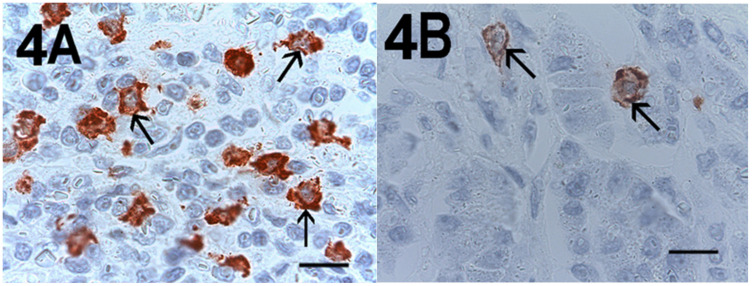
Magnification ×1000 in oil, 0.06 mm^2^ area, immunostaining with the anti-CD117 antibody. (**A**) Very high MCDP-c-KitR in primary PDAT section. Small arrows indicate single red-immunostained MCs. The big arrow indicates tumour epithelium. (**B**) Low MCDP-c-KitR in ANT section. Small arrows indicate single red-stained mast cells. Please note the filiform membranes in immunostaining. Scale bar corresponds to 150 µm.

**Figure 5:**
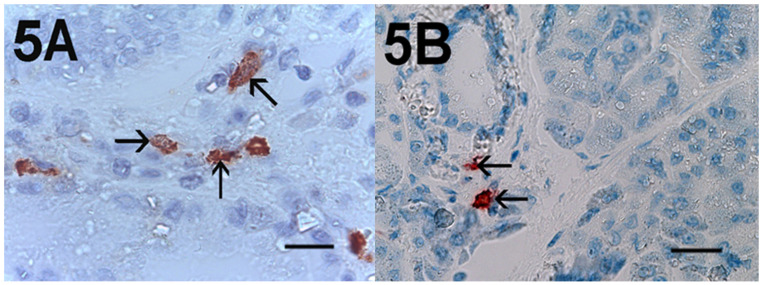
Magnification ×1000 in oil, 0.06 mm^2^ area, immunostaining with the anti-tryptase antibody. (**A**) High MCDPT in primary PDAT section. Small arrows indicate single red-immunostained MCs. Magnification ×1000 in oil. (**B**) Low MCDPT in ANT section. Small arrows indicate red-stained MCs. Scale bar corresponds to 150 µm.

**Figure 6:**
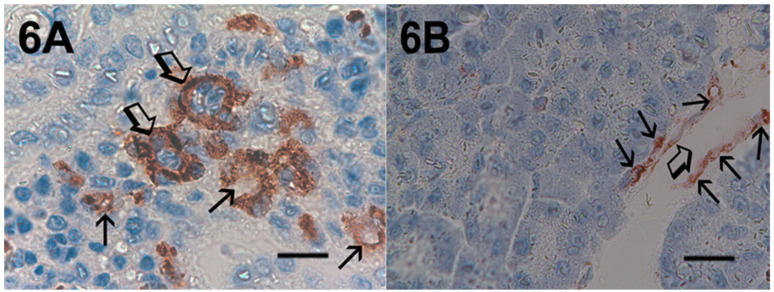
Magnification ×1000 in oil, 0.06 mm^2^ area, immunostaining with the anti-CD31 antibody. (**A**) High MVD in primary PDAT section. Small arrows indicate single red-stained microvessels. Big arrows indicate single microvessels with tumour cells in their lumen. (**B**) Low MVD in ANT section. Small arrows indicate immunostained endothelium of a large vessel; the big arrow indicates the lumen of the vessel. Scale bar corresponds to 150 µm.

Immunohistochemical staining of c-Kit receptor- and tryptase-positive mast cells, besides MVD in PDAT versus ANT, are reported in Figure 1A, Figure 2A, Figure 3A, Figure 1B, Figure 2B or Figure 3B, respectively (×400 magnification), Figure 4A, Figure 5A, Figure 6A, Figure 4B, Figure 5B and Figure 6B respectively (×1000 magnification). 

**Figure 7:**
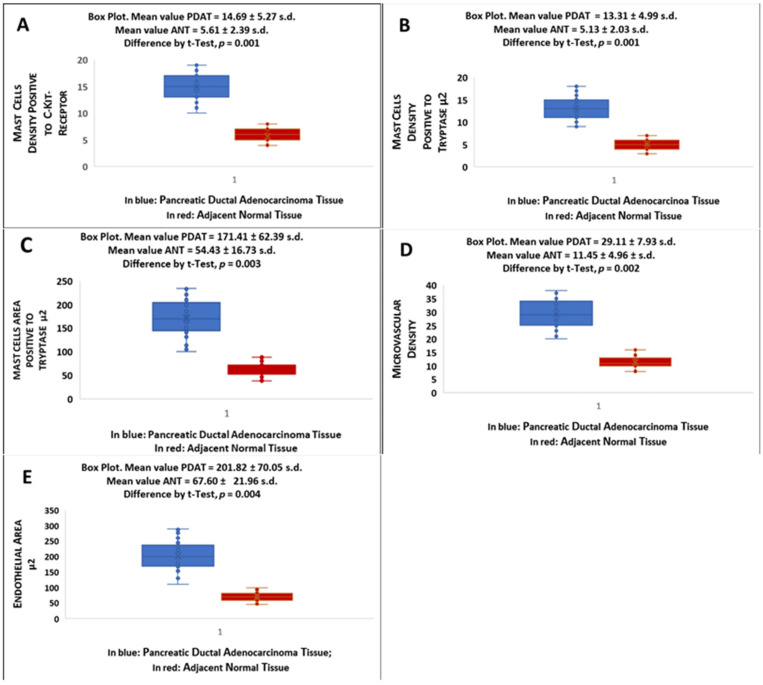
Boxes plot indicate mean ± standard deviation of (**A**) MCDP-c-KitR, (**B**) MCDPT, (**C**) MCAPT, (**D**) MVD and (**E**) EA in PDAT and ANT, respectively, and corresponding differences by Student’s *t*-test in terms of *p*-value.

All results are presented in Figure 7 and the acronyms are reported in the below legend. Data demonstrated that MCDP-c-KitR, MCDPT, MCAPT, MVD and EA significantly increased from ANT to PDAT. Differences in terms of mean value ± SD between PDAT and ANT were significant for each analysed tissue biomarker (*p* ranged from 0.001 to 0.005 by *t*-test analysis). 

**Figure 8:**
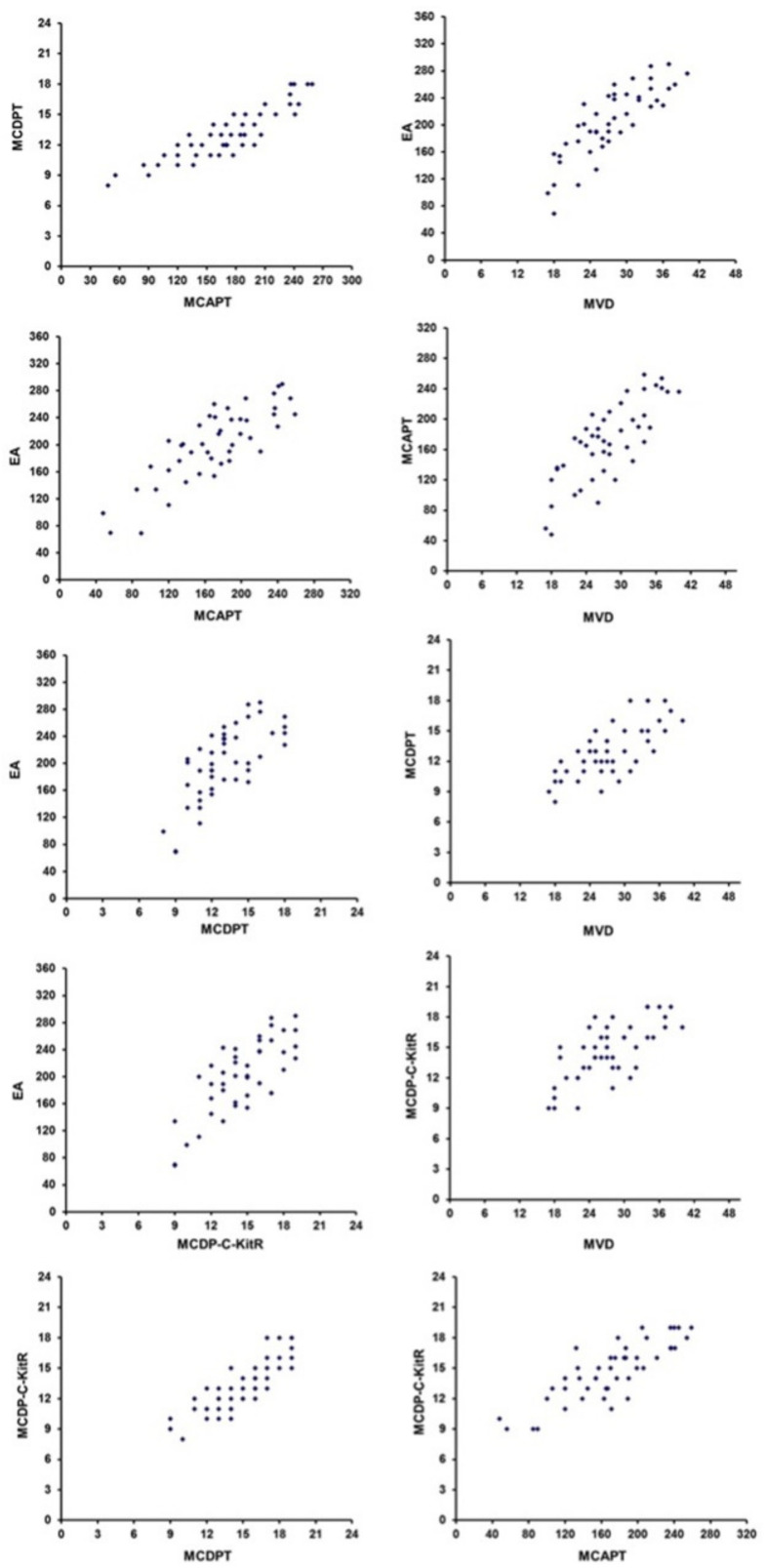
Correlations by Pearson analysis between MCDPT and MCAPT (*r* = 0.85, *p* = 0.01), MVD and EA (*r* = 0.82, *p* = 0.01), EA and MCAPT (*r* = 0.66, *p* = 0.03), MCAPT and MVD (*r* = 0.76, *p* = 0.02), EA and MCDPT (*r* = 0.69, *p* = 0.03), MCDPT and MVD (*r* = 0.72, *p* = 0.02), EA and MCD-c-KitR (*r* = 0.73, *p* = 0.02), MCDP-c-KitR and MVD (r = 0.74, *p* = 0.02), MCDP-c-KitR and MCDPT (r = 0.87, *p* = 0.01) and MCDP-c-KitR and MCAPT (*r* = 0.81, *p* = 0.01).

Statistical evaluation by Pearson analysis in PDAT showed a significant correlation between all parameters, as reported in the caption of Figure 8. 

No other correlation among MCDP-c-KitR, MCDPT, MCAPT, MVD, EA and the main clinical–pathological characteristic was found.

## 3. Discussion

Several published studies suggest that an increased mast cell density is associated with increased MVD in different animal and human malignancies, but to this regard, very little data have been published for PDAT [37,38,39,40,41,42,43,44,45,46,47,48,49,50,51,52,53,54,55,56,57,58]. In the literature, there are some data about the correlation between MVD, evaluated with anti-CD31, and the survival of resected pancreatic cancer patients, demonstrating that high MVD expression is strongly associated with poorer prognosis [59]. At the same time, Strouch et al. [26] showed that the prognosis of resected pancreatic cancer patients with high mast cell count in cancer tissue section, evaluated with anti-tryptase, was significantly worse than those with low mast cell count.

Mast cells migration in the tumour microenvironment is induced by the expression of several growth factors, molecules and proinflammatory cytokines, such as SCF, TGF, TNF, FES kinase, protein kinase D, CXCL12, eicosanoids, chemokines and prostaglandins, and by the activation of subcellular pathways, such as ERβ/CCL2/CCR2, EMT/MMP9 and PI3K/AK. Cells in both the cancer and tumour microenvironment can participate in the production of chemotactic agents for MCs, depending on the tumour type and the specific microenvironment [20,60,61,62,63,64,65]. From a functional point of view, MCs’ activities are mainly regulated by c-Kit-R, known also as CD-117, that binds its ligand SCF, and as a consequence of this interaction, MCs can degranulate [7,25,66,67,68]. It has been well established that MCs can synthesize and then release a plethora of classical proangiogenic factors such as VEGF, FGF-2, sphingosine-1-phosphate (S1P), TNF-α and IL-1, 6 and 8, which may stimulate the proliferation, migration and differentiation of ECs [69,70,71,72]. In particular, S1P synthesis is upregulated upon mast cell activation. Its signalling results in a substantial amount of VEGF-A release and triggers both transcriptional upregulation of VEGF-A and MMP-2 mRNA and protein secretion from mast cells [73]. The proangiogenic effect of MCs is also related to the production of gelatinase A (matrix metalloproteinase-2) and gelatinase B (matrix metalloproteinase-9) that degrade the extracellular matrix, releasing the prestored VEGF that, in turn, stimulates EC proliferation [74,75,76]. MCs also synthesize tryptase, a non-classical potent proangiogenic factor, which represents the most abundant biological substance in MCs’ secretory granules [14,15]. From a biological point of view, tryptase is a trypsin-like neutral serine protease and selective component of the secretory granules of all human MCs, accounting for almost 25% of cell protein (10–35 pg per mast cell) [15]. It consists of four monomers, each of which is stabilized in its active conformation and its tetrameric form by heparin–proteoglycan complexes [20,77,78]. Because of its proteolytic activity, tryptase acts as an agonist of the protease-activated receptor-2 (PAR-2), a G protein expressed on ECs that is involved in their proliferation and whose activation triggers an intracellular signal involving MAPK phosphorylation pathway [79,80].

Tryptase may also convert pro-MMP to active MMP, enhancing the degradation of extracellular matrix, VEGF release and neoangiogenesis. Chymase plays this role too [81,82].

The milestone in vitro data stating mast cell tryptase’s capacity to induce microvessel formation was first presented by Blair in 1997 [16]. In this study, a coculture of the human mast cells-1 (HMC-1) line with their products in the presence of human dermal microvascular ECs led to vessel formation, and the extent of neovascularization was strongly enhanced when HMC-1 MCs were degranulated. The results of this study indicated that tryptase from degranulated MCs was able to induce the genesis of microvessels with an increasing dose–response pattern. To support these findings, the inhibition of tryptase through a specific tryptase inhibitor led to a strong reduction of microvasculature formation. More recently, they demonstrated the angiogenic properties of MC tryptase in an in vivo chorioallantoic membrane assay, showing that the angiogenic potency of tryptase was similar to VEGF [11,17].

With particular reference to pancreatic cancer, preclinical in vivo data suggest that mast cell tryptase plays a role in stimulating angiogenesis and cancer growth also via the activation of the proangiogenic factor angiopoietin-1 [35,83,84]. Based on this biological background, in a previously published pilot study, we evaluated the correlation among MCDPT, MCAPT, MVD and EA in a series of 31 patients with resected primary pancreatic cancer [23]. Results from our study showed an evident association among these parameters, suggesting that tryptase-positive mast cells and the magnitude of tryptase expression correlate well with both MVD and EA in pancreatic cancer angiogenesis. Nevertheless, in this study, no ANT was evaluated and no MCDP-c-KitR was assessed. In a subsequent study conducted in a series of 35 PDAT patients, we observed a significant correlation between MCDP-c-KitR, MCDPT and MVD, and in particular, an increased MCDPT and MVD were found in PDAT when compared to ANT, confirming the role of mast cell tryptase in pancreatic cancer angiogenesis and tumour development [25]. In the latter study, however, no evaluation of MCAPT and EA was performed in normal and cancer tissues.

In the present exploratory research, as innovative technical approaches, immunohistochemistry and an image analysis system allowed us to access MCDP-c-KitR, MCDPT, MCAPT, MVD and EA simultaneously, both in PDAT and ANT, in a series of patients who had undergone radical surgery. Endothelial area represents the immunostained vascular area in a microscopic field, which is independent of their capacity and diameter, and can be interpreted as a surrogate of angiogenic activity in parallel; MCDPT indicates the number of local mast cells and not the area in which the mast cells are acting. We maintain that the identification of a couple of parameters (area and density) is more representative of the real angiogenic (EA and MVD) and enzymatic (MCDPT and MCAPT) activities. Our data indicated not only that MCs accumulate in pancreatic cancer tissue, but also MCAPT is increased in PDAT. The strong correlation between MCDP-c-KitR and MCDPT also suggests that in pancreatic cancer, the majority of MCs are tryptase-expressing cells. As expected, these data strongly correlate to each other and represent internal controls for the pancreatic disease. Nevertheless, to our knowledge, this is the first study that clearly shows these correlations through a new integrated immunohistochemical and image analysis system.

Our data are also supported by Chang’s research involving a transgenic spontaneous PDAT C57BL/6 mouse model, in which an early influx of MCs into the tumour microenvironment was assessed [33]. Even more interestingly, the growth of PDAT was significantly suppressed in mast cell-deficient *Kit^w-sh/w-sh^* mice, demonstrating the importance of MCs and their activation by c-Kit receptor in leading to angiogenesis and pancreatic cancer development. Moreover, Gorzalczany et al. showed that cell-to-cell contact interactions by exposing MCs to membranes derived from cancer cells resulted in mast cell activation, leading to increased phosphorylation of the ERK1/2 MAP kinases and Akt through a phosphatidylinositol 3-kinase-dependent pathway [85]. These in vitro results indicated a further mechanism of mast cell activation in tumour stromal microenvironments. 

All preclinical data support the results of our investigation, whose message indicates increased tryptase expression parallel to increased angiogenesis in terms of both MVD and higher EA extension in surgically treated PDAT patients. 

## 4. Conclusions

Here, we speculate that MCDPT and MCAPT together could be putative tissue biomarkers of pancreatic cancer angiogenesis status. To this regard, tryptase targeting through tryptase inhibitors, such as gabexate or nafamostat mesylate, could become an interesting strategy as a novel antiangiogenetic intervention in pancreatic cancer patients [86,87,88,89,90]. On the other hand, MC degranulation could be inhibited by c-KitR tyrosine kinase inhibitors, such as masitinib, as first applied in veterinary clinical oncology and then translated to humans for the treatment of PDAT patients, with interesting results, as reported by the only phase 3 clinical trial [91,92,93,94,95]. Finally, MCDPT and MCAPT could become potential predictive biomarkers of response to anti-c-Kit or anti-tryptase therapy, in order to to select patients with a higher risk as assessed by these biomarkers [96]. Confirmatory study and clinical trials are awaited in this context as well as novel anti-angiogenic therapies. 

## 5. Materials and Methods 

### 5.1. Study Population

The clinical–pathological characteristics of the analysed patients are summarized in Table 1. A total of 45 PDAT patients with stage T_2–3_N_0–1_M_0_ amenable to surgery underwent potentially curative resection. Employed surgical procedures were pancreaticoduodenectomy, distal pancreatectomy and total pancreatectomy with lymph node dissection [25]. Patients were staged according to the American Joint Committee on Cancer 7th edition (AJCC-TNM) classification, and the World Health Organization classification (2000 version) was used for pathological grading. All patients had no distant metastases on computed tomography. Full ethical approval and signed consent from individual patients were obtained. The study was conducted in accordance with the Declaration of Helsinki, and the protocol was approved by the Ethics Committee of the “Mater Domini” Hospital, “Magna Graecia” University, Catanzaro (No. 242; 22 December 2016).

### 5.2. Immunohistochemistry

Both MCs positive for c-KitR and tryptase and vessels were assessed by immunohistochemistry, employing a three-layer biotin–avidin–peroxidase technique [23,24,25,43]. In summary, 5 μm-thick serial sections of formalin-fixed and paraffin-embedded PDAT and ANT were cut. The obtained slides were processed with a microwave oven at 500 watts for 10 min, and then the endogenous peroxidase enzyme was inhibited with a 3% hydrogen peroxide solution. Soon after, the slides were incubated with the following primary antibodies:

Anti-CD117 to c-KitR (Dako, Glostrup, Denmark) diluted 1:100 at for 1 h at room temperature;

Anti-tryptase (clone AA1; Dako, Glostrup, Denmark) diluted 1:100 for 1 h at room temperature (for MC identification);

Anti-CD31 antibody (QB-END 10; Bio-Optica Milan, Milan, Italy) diluted 1:50 for 1 h at room temperature (as a pan-endothelial marker).

Immunoreactivity was evidenced by employing a biotinylated secondary antibody, the red chromogen avidin–biotin–peroxidase complex (LPS, K0640, Dako, Glostrup, Denmark). Cell nuclei were stained with Gill′s haematoxylin no. 2 (Polysciences, Warrington, PA, USA). No primary antibody was posted in negative controls.

### 5.3. Morphometrical Assay 

A light microscope integrated with an image analysis system (AXIO, Scope A1, ZEISS, Germany) was utilized. For each serial section of PDAT and ANT, the five most heavily immunostained areas (hot spots) were selected at low magnification. Next, MCDP-C-KitR, MCDPT, MCAPT, MVD and EA were assessed at ×400 magnification (0.19 mm^2^ area) in the five identified hot spot areas for each serial section, respectively (Figure 1A,B, Figure 2A,B, Figure 3A,B). With special reference to MCDP-c-KitR and MCDPT, each immunostained cell was considered in the count, whereas MVD was detected by counting single red-brown-stained endothelial cells, endothelial cell clusters and microvessels, clearly separated from adjacent microvessels, according to the modified Weidner’s method [23,24,25]. Immunostained MCs positive for tryptase and endothelial cells positive for anti-CD31 were also evaluated in terms of immunostained area at ×400 magnification (0.19 mm^2^ area). Finally, morphological details of MCs positive for c-KitR, MCs positive for tryptase and endothelial cells were observed at ×1000 magnification in oil (Figure 4A,B, Figure 5A,B, Figure 6A,B, respectively). 

### 5.4. Statistical Analysis

Mean value for each section and for the global series was obtained for all evaluated parameters in both PDAT and ANT groups. The differences between the two groups were measured by Student’s *t*-test. Mean values ± 1 standard deviation (SD) of all evaluated tissue parameters are reported in Figure 7.

Correlations between MCDP-c-KitR, MCDPT, MCAPT, MVD and EA were calculated using Pearson′s (*r*) analysis (Figure 7). Correlations among all studied parameters and the most important clinical–pathological characteristics, reported in Table 1, were analysed by the chi-square test (χ^2^). All analyses were considered statistically significant with a *p* value of < 0.05. Statistical analyses elaboration was performed with the SPSS statistical software package (SPSS, Inc., Chicago, IL, USA). 

## Figures and Tables

**Table 1:** Clinical–pathological features of patients (*n* = 45).

Subgroup	No. of Patients
Age	
< 65	30 (67%)
> 65	15 (33%)
Gender:	
Female	23 (51%)
Male	22 (49%)
Tumour site:	
Head	19 (42%)
Body-Tail	26 (58%)
TNM by AJCC Stage	
T_2_N_0–1_M_0_	20 (44%)
T_3_N_0–1_M_0_	25 (56%)
Histologic type	
Ductal adenocarcinomas	45 (100%)
Histologic grade	
G1–G2	34 (77%)
G3	11 (23%)

## Data Availability

All relevant data are included in the article.
